# LungMicroHostR: an R package for integrated host–microbiome analysis of bronchoalveolar lavage fluid metagenomic sequencing data

**DOI:** 10.3389/fbinf.2026.1906036

**Published:** 2026-08-10

**Authors:** Nan Li, Jing Hu, Wanning Tong, Chengdong Liu, Yun Ding, Ning Li, Zhigang Cai

**Affiliations:** 1 Department of Cardiothoracic Surgery, Naval Medical Center, Naval Medical University, Shanghai, China; 2 Department of Respiratory Medicine, Naval Medical Center, Naval Medical University, Shanghai, China

**Keywords:** bronchoalveolar lavage fluid, host–microbiome analysis, lung cancer, negative controls, R package, respiratory metagenomics

## Abstract

**Introduction:**

Bronchoalveolar lavage fluid metagenomic next-generation sequencing captures microbial profiles and host-derived molecular measurements from the same respiratory specimen, but downstream analysis requires coordinated handling of low-biomass microbial signals, negative-control information and multiple feature tables.

**Methods:**

We developed LungMicroHostR, an R package for downstream host–microbiome analysis of bronchoalveolar lavage fluid metagenomic sequencing data. The package brings processed microbial profiles, host-derived molecular measurements, sample metadata and negative-control information into a unified R workflow for feature filtering, comparative model evaluation, visualization and reproducible reporting.

**Results:**

Using the public GSE252118 resource comprising 402 samples from patients with lung cancer or pulmonary infections, LungMicroHostR assembled matched microbial, host and clinical feature tables, estimated prevalence in negative controls and compared host transcriptomic, microbial-profile and combined host–microbial models. In the test set, the 10-feature host transcriptome nearest-centroid model achieved an AUC of 0.772 (95% confidence interval, 0.680–0.860), the five-feature RNA microbial logistic model achieved an AUC of 0.745 (0.655–0.832), and the combined host transcriptome–RNA microbial logistic model achieved an AUC of 0.765 (0.655–0.866) with balanced accuracy of 0.720. An external PRJNA714488 BALF shotgun metagenomic dataset was additionally analysed at the mOTU level; LungMicroHostR matched the resulting feature table with phenotype metadata and generated a 388-feature by 26-sample microbial abundance matrix.

**Discussion:**

LungMicroHostR provides documented functions for respiratory metagenomic analyses that require joint evaluation of microbial profiles, host-derived measurements, negative-control information and external microbial feature tables.

## Introduction

Bronchoalveolar lavage fluid (BALF) metagenomic next-generation sequencing (mNGS) has broadened respiratory disease research by enabling microbial nucleic acids and host molecular features to be profiled from the same clinical specimen (Han et al., 2025; Tang et al., 2025). In lung cancer and pulmonary infection, processed BALF mNGS resources can include microbial composition, host transcriptional profiles, immune-cell estimates, copy number variation summaries and tumour fraction estimates, extending the analysis beyond microbial abundance alone (Han et al., 2025; Tang et al., 2025). The combination of microbial and host-derived measurements calls for reproducible workflows that keep feature tables, sample metadata and negative-control information connected during downstream analysis (Beghini et al., 2021; Tang et al., 2025).

Microbiome analysis workflows integrate taxonomic and functional profiling of sequencing data with statistical analysis and visualization of processed feature tables (Beghini et al., 2021). A broad ecosystem of web applications, R packages and workflow-oriented platforms has been developed for downstream microbiome analysis (McMurdie and Holmes, 2013; Buza et al., 2019; Shanmugam et al., 2021; Liu et al., 2021; Liu et al., 2022; Lu et al., 2023; Xu et al., 2023; Liu et al., 2026). However, BALF mNGS datasets require joint analysis of microbial profiles, host-derived molecular features, sample metadata and negative-control information within a common workflow.

Lower-airway specimens may contain low microbial biomass relative to background nucleic acids, and microbial profiles from low-biomass lower-airway materials can be influenced by reagent-derived DNA, cross-contamination and other background signals (Salter et al., 2014; Eisenhofer et al., 2019). Negative controls provide important context for interpreting microbial features, particularly when low-abundance taxa are included in statistical analysis (Davis et al., 2018; Eisenhofer et al., 2019; Fierer et al., 2025). For BALF mNGS data, prevalence estimates from negative controls therefore need to be considered alongside feature tables and sample metadata during downstream analysis.

Integrated host–microbiome analysis adds further complexity because microbial abundance tables, host transcriptomic measurements, immune-cell estimates and clinical annotations are generated as separate data layers and must be matched at the sample level before statistical analysis and visualization (Deek et al., 2024). A downstream workflow for BALF mNGS data therefore needs to keep microbial feature tables, host-derived molecular measurements, sample metadata and negative-control information aligned across data integration, statistical analysis and visualization.

Published analyses of lung cancer-associated respiratory microbiota have reported associations between microbial profiles and lung cancer or pulmonary disease states, with microbial patterns varying by sampling route, sample biomass, experimental design and contamination-control procedures (Zheng et al., 2021; Cheng et al., 2024; McElderry et al., 2026). Evidence from respiratory microbiota analysis also points to the need for transparent reporting of feature filtering, negative-control information and model-performance summaries (Davis et al., 2018; Eisenhofer et al., 2019; Fierer et al., 2025; McElderry et al., 2026).

Machine learning models have increasingly been used in microbiome and metagenomic analysis. Supervised classifiers can compare microbial profiles, host-derived molecular measurements and combined feature sets according to their ability to separate disease groups. In BALF mNGS data, model comparison can evaluate whether microbial profiles provide information beyond host transcriptomic features and whether combined feature sets improve classification of lung cancer and pulmonary infection (Wirbel et al., 2021; Topçuoğlu et al., 2021; Deek et al., 2024).

Here we present LungMicroHostR, an R package for downstream analysis of BALF mNGS host–microbiome data. Its specific contribution is a BALF-oriented analysis record that keeps processed microbial and host-derived feature tables, sample metadata, negative-control flags, training and test partitions, source-specific model comparisons, calibration and resampling summaries, and figure-ready result tables connected within one workflow. The package accepts microbial feature tables across taxonomic or feature levels, including genus-level and mOTU-level profiles, and was evaluated using the GSE252118 BALF mNGS resource together with an external PRJNA714488 BALF shotgun metagenomic dataset.

## Methods

### Software implementation and package design

LungMicroHostR was implemented as an R package for downstream analysis of BALF mNGS datasets that contain microbial profiles and host-derived molecular features. The package is positioned downstream of read-level quality control and taxonomic profiling and operates on processed feature tables generated by metagenomic, metatranscriptomic and host transcriptomic workflows. Read-level preprocessing and profiler selection are completed upstream, after which LungMicroHostR performs sample harmonisation, feature-table analysis, comparative model evaluation, visualization and reproducible reporting.

**FIGURE 1:**
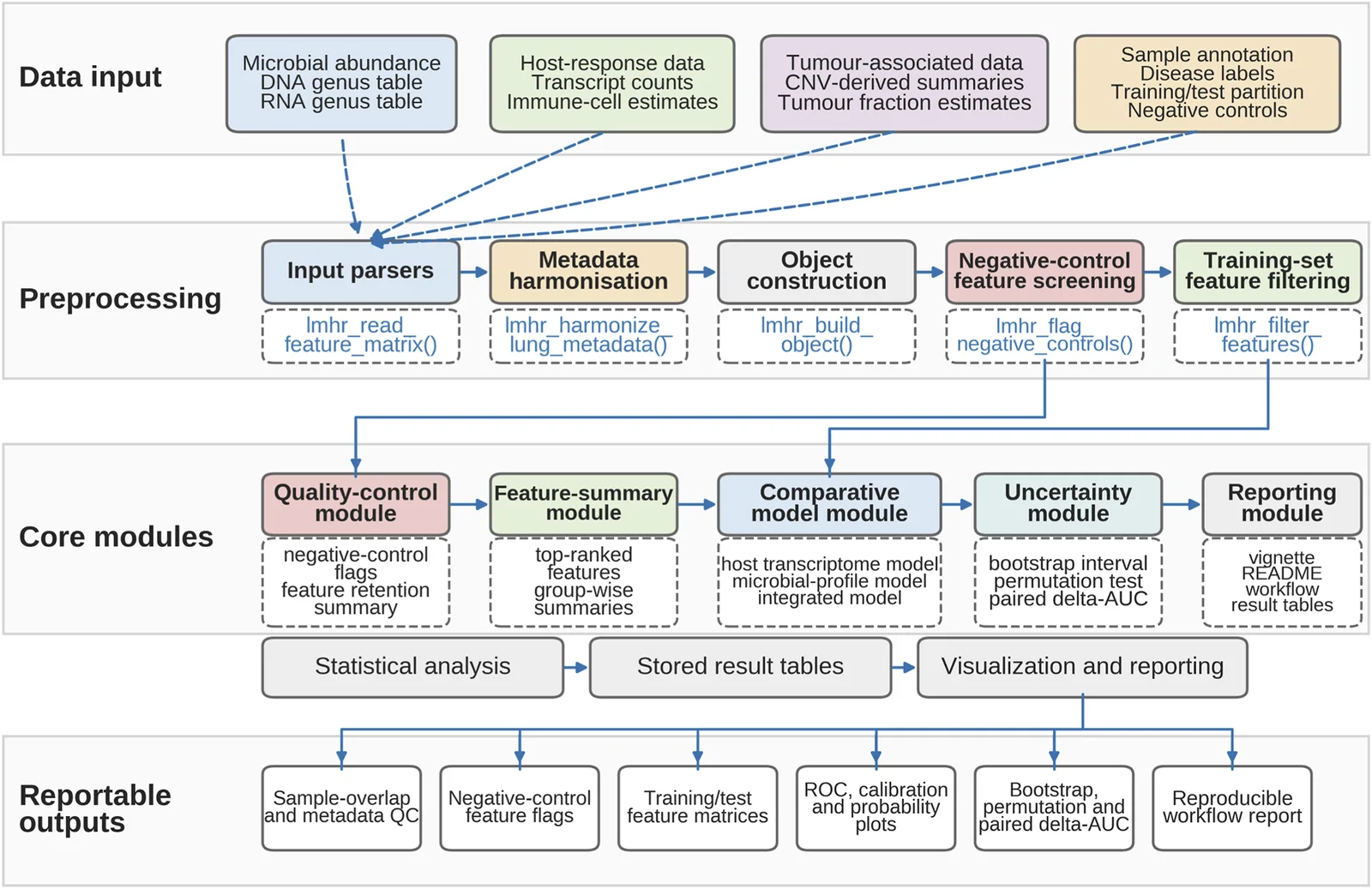
Overview of LungMicroHostR. The input layer comprises processed microbial feature tables, host-response and tumour-associated feature tables, sample annotations, predefined data partitions and negative-control tables. Preprocessing functions harmonise metadata, construct the analysis object and generate screening and filtering records; downstream modules produce feature summaries, comparative model evaluation, uncertainty estimates and reusable reports. Solid arrows show the primary analysis flow, whereas dashed arrows and boxes show optional or function-level operations.

The package design contains four components: data input, preprocessing, analysis modules and result reporting (Figure 1). The input component accepts microbial feature tables at genus, species or mOTU levels, host transcript count matrices, immune-cell proportion estimates, copy number variation (CNV)-derived summaries, sample annotations, disease labels, training and test partitions and negative-control tables. The preprocessing component harmonises metadata, constructs analysis objects and records feature-filtering decisions. The analysis modules summarize microbial features, compare feature-source-specific models, estimate resampling-based uncertainty and generate reusable result tables.

Representative functions read feature matrices, harmonise disease-state labels, construct a data object, record features with high prevalence in negative controls, filter features using training data, calculate classification performance metrics, estimate bootstrap intervals and write result tables. Statistical result tables are written before visualization so that feature tables, model performance summaries and figures can be inspected and regenerated from the same intermediate and final outputs.

### Public BALF mNGS dataset and data integration

The workflow was evaluated using the public GSE252118 BALF mNGS resource, which provides processed microbial and host molecular profiles for patients with lung cancer or pulmonary infections (Han et al., 2025; Tang et al., 2025). The resource includes DNA microbial abundance profiles, RNA microbial abundance profiles, host RNA sequencing counts, immune-cell proportion estimates, CNV-derived summaries and tumour fraction estimates, sample-level disease annotations, predefined training and test partitions and count tables for negative controls. All analyses were performed on processed feature tables and metadata tables available from the public resource.

**FIGURE 2:**
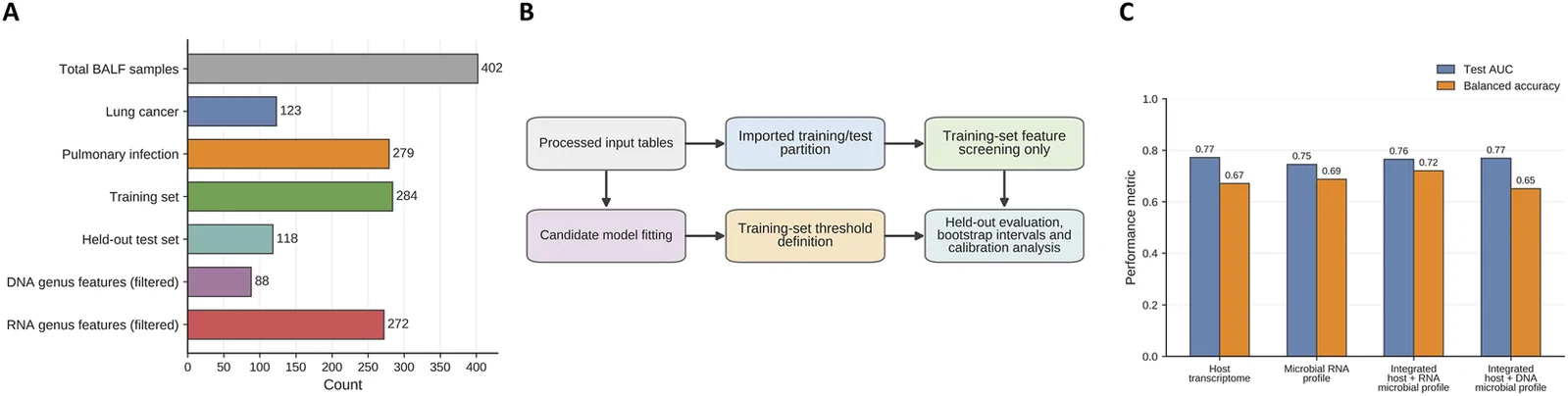
GSE252118 BALF mNGS dataset and model evaluation. **(A)** Composition of the 402-sample dataset and numbers of DNA and RNA genus features retained after negative-control-based screening. **(B)** Analysis flow from processed input tables through training-set feature screening, model fitting and threshold definition to test-set discrimination, bootstrap uncertainty and calibration. **(C)** Test-set AUC and balanced accuracy for representative host transcriptome, RNA microbial and integrated host–microbial models; bar colour identifies the performance metric.

A total of 402 BALF samples were included in the binary lung cancer versus pulmonary infection analysis, comprising 123 lung cancer samples and 279 pulmonary infection samples. The infection group included bacterial infection, fungal infection and pulmonary tuberculosis annotations. The predefined binary partition contained 284 training samples and 118 test samples, and the same partition was preserved throughout the analysis. Feature ranking, scaling, model fitting and threshold definition were performed in the training set, whereas final model performance evaluation and resampling-based uncertainty estimation were performed in the test set (Figure 2).

Processed matrices were aligned to sample metadata by sample identifiers. The final data tables contained 402 matched samples across the DNA microbial profile, RNA microbial profile, host transcript count matrix, immune-cell proportion matrix and CNV-derived matrix. Count tables for negative controls were kept as separate inputs and used to calculate prevalence in negative controls before feature filtering.

### Negative-control-based feature screening and feature tables for model fitting

BALF is a lower-airway respiratory specimen in which microbial profiles can be influenced by background signals. For each genus-level feature, prevalence and mean relative abundance were calculated from training samples, and prevalence in negative controls was calculated from the corresponding negative-control count table. We refer to this step as negative-control-based feature screening: it records features that recur in negative controls and generates transparent screening flags for downstream analysis. This procedure is distinct from formal contamination-correction models that estimate contaminant probabilities from prevalence or frequency patterns (Davis et al., 2018).

**FIGURE 3:**
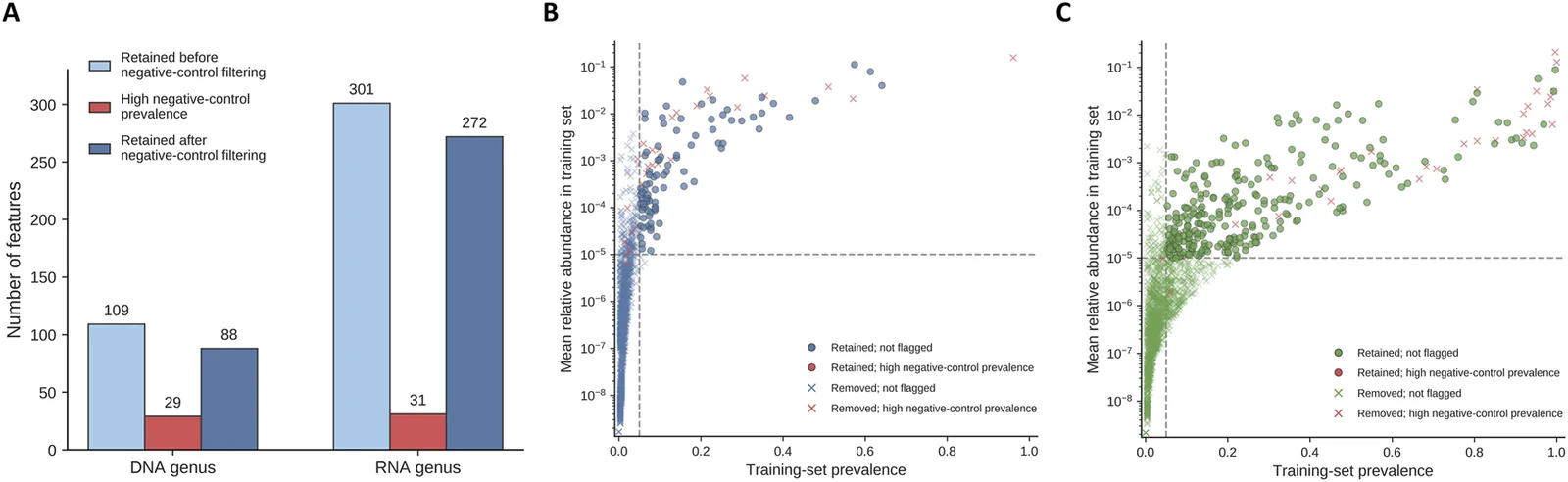
Negative-control-based microbial feature screening. **(A)** Numbers of DNA and RNA genus features retained before negative-control filtering, flagged for high negative-control prevalence and retained after filtering. **(B)** Training-set prevalence and mean relative abundance of DNA genus features. **(C)** Corresponding RNA genus features. Circles denote retained features and crosses denote removed features; red symbols indicate high prevalence in negative controls, and dashed lines indicate the prespecified training-set prevalence and mean-abundance thresholds.

Features were retained for subsequent analysis if they had a prevalence of at least 0.05 in the training set and a mean relative abundance of at least 1 × 10^−5^ in the training set. These thresholds were prespecified for the case study to remove extremely rare or very low-abundance features that can yield unstable estimates in sparse microbiome data, while preserving a broad candidate set. Features with prevalence greater than 0.10 in negative controls were recorded as recurrent negative-control features and excluded from the negative-control-filtered feature tables after the training-set criteria were applied (Figure 3). The numerical cutoffs were not optimized on the test set; their use follows the broader recommendation that negative controls and prevalence-based screening should be incorporated into low-biomass microbiome analyses (Salter et al., 2014; Davis et al., 2018; Eisenhofer et al., 2019).

Host RNA sequencing counts were transformed into log-count-per-million values after library-size normalization. Host transcriptomic features were ranked in the training set according to their association with the binary disease label. Representative low-dimensional candidate models used prespecified top-five microbial and top-10 or top-20 host feature sets, depending on the data layer. Feature identities were ranked using the training data only, and the test set was not used to select feature identities, feature-set size, centring or scaling parameters. Feature tables for model fitting were generated for microbial profiles, host transcriptomic features, immune-cell estimates, CNV-derived summaries and combinations of host and microbial features.

### Model performance evaluation and resampling-based uncertainty estimates

The modeling module in LungMicroHostR is intended primarily for comparative model evaluation across biological feature sources. It provides a common supervised-learning benchmark for quantifying discrimination, threshold-based classification, calibration and uncertainty for microbial profiles, host transcriptomic measurements, immune-cell estimates, CNV-derived summaries and host–microbial combinations. Low-dimensional feature sets were analysed with logistic-regression models, selected host and combined feature sets were evaluated with nearest-centroid classifiers, and higher-dimensional feature sets were assessed using principal-component logistic regression.

For each model, the classification threshold was determined in the training set by maximizing the Youden index and was then applied to the test set. Test-set performance was summarized using the area under the receiver operating characteristic curve (AUC), average precision, accuracy, balanced accuracy, sensitivity, specificity, positive predictive value, negative predictive value, F1 score and Brier score.

Uncertainty was estimated using bootstrap resampling of the test samples. For selected models, 1,000 bootstrap resamples were used to estimate 95% confidence intervals for AUC and other performance metrics. Permutation testing evaluated the observed AUC relative to the distribution obtained under label exchangeability. Paired bootstrap resampling estimated AUC differences between selected models. Calibration profiles were obtained by grouping test-set predictions into probability bins and comparing mean predicted probabilities with observed lung cancer proportions.

### External BALF mOTU-level feature-table evaluation

PRJNA714488 was used as an external application example for BALF microbial-profile feature-table analysis. The dataset contained 26 BALF samples with phenotype annotation, including 10 controls and 16 non-small cell lung carcinoma samples. Raw paired-end sequencing files were profiled using mOTUs v4 to generate mOTU-level taxonomic profiles (Milanese et al., 2019). Assigned mOTU counts were extracted from each profile, unassigned entries were removed and sample-wise relative abundance values were calculated from assigned counts.

The resulting mOTU-level feature table was imported into LungMicroHostR and matched with phenotype metadata. LungMicroHostR was then used to summarize sample-level microbial assignment, feature prevalence, alpha diversity, Bray-Curtis principal coordinate analysis, exploratory feature-level group comparisons and exploratory repeated stratified cross-validation. The corresponding feature matrix, metadata table, ordination coordinates and model-evaluation outputs were retained as reproducible analysis files.

## Results

### Modular BALF mNGS analysis workflow

LungMicroHostR was applied to processed BALF mNGS data containing microbial profiles, host-derived molecular features, sample annotations and negative controls. The package assembled the input tables into a common data object and generated sample matching summaries, feature filtering tables, prevalence estimates from negative controls, feature tables for model fitting and model performance tables (Figure 1).

### Public BALF mNGS dataset

The public BALF mNGS dataset included 402 samples, comprising 123 lung cancer samples and 279 pulmonary infection samples. The infection group included bacterial infection, fungal infection and pulmonary tuberculosis. After sample matching, DNA microbial profiles, RNA microbial profiles, host transcriptomic data, immune-cell estimates and CNV-derived summaries were available for the same 402 samples (Figure 2A).

The predefined binary partition contained 284 training samples and 118 test samples (Figure 2B). This predefined partition was used to compare models based on microbial profiles, host transcriptomic features, immune-cell estimates, CNV-derived summaries and combinations of host and microbial features under the same evaluation procedure (Figure 2C).

### Negative-control-based feature screening

Before feature filtering, the DNA microbial profile contained 1,500 genus-level features and the RNA microbial profile contained 1,630 genus-level features. Training-set prevalence and abundance criteria retained 109 DNA genus features and 301 RNA genus features before features with high prevalence in negative controls were excluded. The screening step recorded 29 DNA genus features and 31 RNA genus features with high prevalence in negative controls. Exclusion of features with high prevalence in negative controls left 88 DNA genus features and 272 RNA genus features for subsequent analysis (Figure 3A).

Scatter plots of training-set prevalence and mean relative abundance showed that many microbial genera had low prevalence or low abundance, particularly among features removed by the filtering criteria (Figures 3B,C).

Model results were organized by feature source. The modeling module was used as a common comparison framework for host transcriptomic, microbial and combined host–microbial feature tables, with test-set discrimination, calibration and resampling-based uncertainty reported under the same procedure.

### Host transcriptomic and microbial-profile models

**FIGURE 4:**
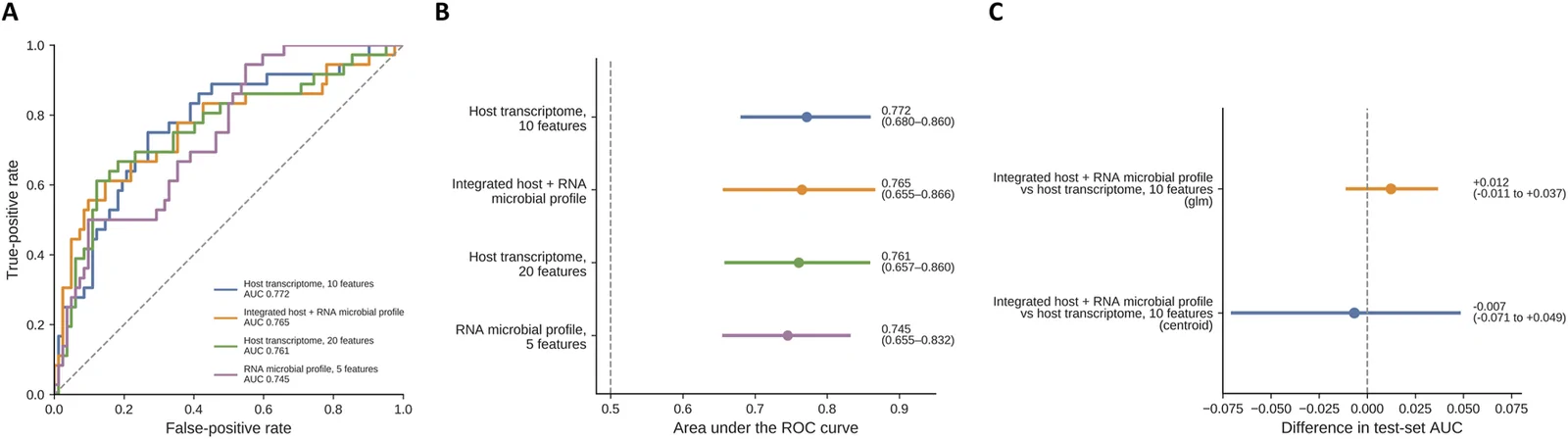
Comparative test-set model performance. **(A)** Receiver operating characteristic curves for representative host transcriptome, RNA microbial and integrated host–microbial models; curve labels report test-set AUC. **(B)** Observed test-set AUCs with 95% confidence intervals from 1,000 bootstrap resamples, which summarize uncertainty around the scalar AUC estimates shown by the full ROC profiles in panel **(A-C)** Paired-bootstrap AUC differences between selected model pairs; positive values favour the first model named, and the dashed vertical line marks zero difference.

In the test set, models based on host transcriptomic features showed the strongest performance for distinguishing lung cancer from pulmonary infection. The host transcriptome model using 10 top-ranked features and a nearest-centroid classifier achieved the highest selected test-set AUC (0.772; 95% confidence interval, 0.680–0.860), with balanced accuracy of 0.671, sensitivity of 0.500, specificity of 0.841 and Brier score of 0.173 (Figures 4A,B). A host transcriptome model using 20 top-ranked features achieved a similar AUC (0.761; 95% confidence interval, 0.657–0.860) and the highest balanced accuracy among the selected models (0.731). Figure 4A displays threshold-dependent discrimination across the complete ROC profiles, whereas Figure 4B summarizes resampling uncertainty around the corresponding AUC estimates.

RNA microbial features also retained measurable discriminatory signal. The RNA microbial model using five top-ranked genus features and logistic regression achieved a test-set AUC of 0.745 (95% confidence interval, 0.655–0.832). The RNA microbial model showed high specificity (0.902) and lower sensitivity (0.472). DNA microbial models and CNV-derived models showed lower test-set performance than the leading host transcriptome and RNA microbial models in the selected model summaries.

### Integrated models

Integrated models evaluated microbial features in combination with host transcriptomic features. The logistic-regression model combining 10 host transcriptomic features with five RNA microbial genus features achieved a test-set AUC of 0.765 (95% confidence interval, 0.655–0.866), balanced accuracy of 0.720, sensitivity of 0.611, specificity of 0.829 and the lowest Brier score among the selected models (0.168). Relative to the strongest host transcriptome model, the combined host transcriptome and RNA microbial model showed a more balanced sensitivity/specificity profile and comparable calibration.

Paired bootstrap comparisons showed small AUC differences between selected host-only and combined models (Figure 4C). The AUC difference between the logistic-regression model combining host transcriptomic and RNA microbial features and the host transcriptome nearest-centroid model was −0.007, with a 95% bootstrap interval crossing zero. The AUC difference between the same combined model and the host transcriptome logistic model was 0.012, with a 95% bootstrap interval also crossing zero.

### Predicted probabilities, calibration curves and coefficient profiles

**FIGURE 5:**
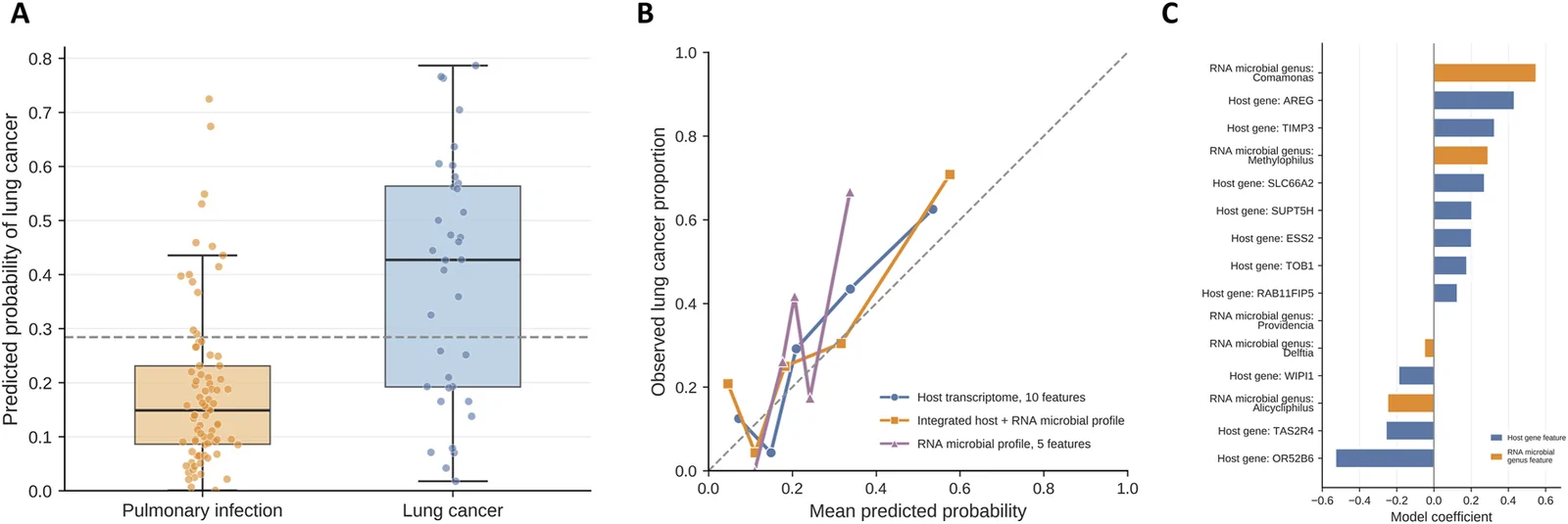
Predictions, calibration and coefficients from the integrated host–RNA microbial logistic-regression model. **(A)** Test-set predicted probabilities for pulmonary infection and lung cancer; points represent samples, boxes summarize group distributions, and the dashed line marks the training-set classification threshold. **(B)** Calibration profiles for the host transcriptome, RNA microbial and integrated models; the diagonal indicates perfect agreement between mean predicted probability and observed lung cancer proportion. **(C)** Coefficients from the integrated model combining 10 host transcriptomic and five RNA microbial genus features; blue and orange bars indicate host and microbial features, respectively, and coefficient sign indicates direction of association with the predicted lung cancer probability.

Predicted probabilities from the logistic-regression model combining host transcriptomic and RNA microbial features were higher overall in lung cancer samples than in pulmonary infection samples, while the two groups showed partially overlapping probability distributions (Figure 5A). The calibration profile showed an increasing observed lung cancer proportion across the upper probability strata, rising from 0.250 at a mean predicted probability of 0.182–0.304 at 0.316 and 0.708 at 0.576 (Figure 5B). The fourth bin was close to the diagonal reference line, whereas the highest-probability bin showed a higher observed lung cancer proportion than the corresponding mean predicted probability. Lower probability strata showed larger fluctuations in observed event proportions.

The coefficient profile of the logistic-regression model combining host transcriptomic and RNA microbial features included both host transcriptomic features and RNA microbial genus features (Figure 5C). Among the positive coefficients, the largest values were observed for the RNA microbial genus Comamonas, the host transcriptomic features AREG and TIMP3, the RNA microbial genus Methylophilus, and the host transcriptomic feature SLC66A2. The largest negative coefficients were observed for the host transcriptomic feature OR52B6, followed by TAS2R4, the RNA microbial genus Alicycliphilus, WIPI1 and the RNA microbial genus Delftia.

Together, the predicted probability distributions, calibration curves and coefficient profiles linked selected host and microbial features with fitted model scores and model interpretation.

### External BALF mOTU-level feature-table evaluation

**FIGURE 6:**
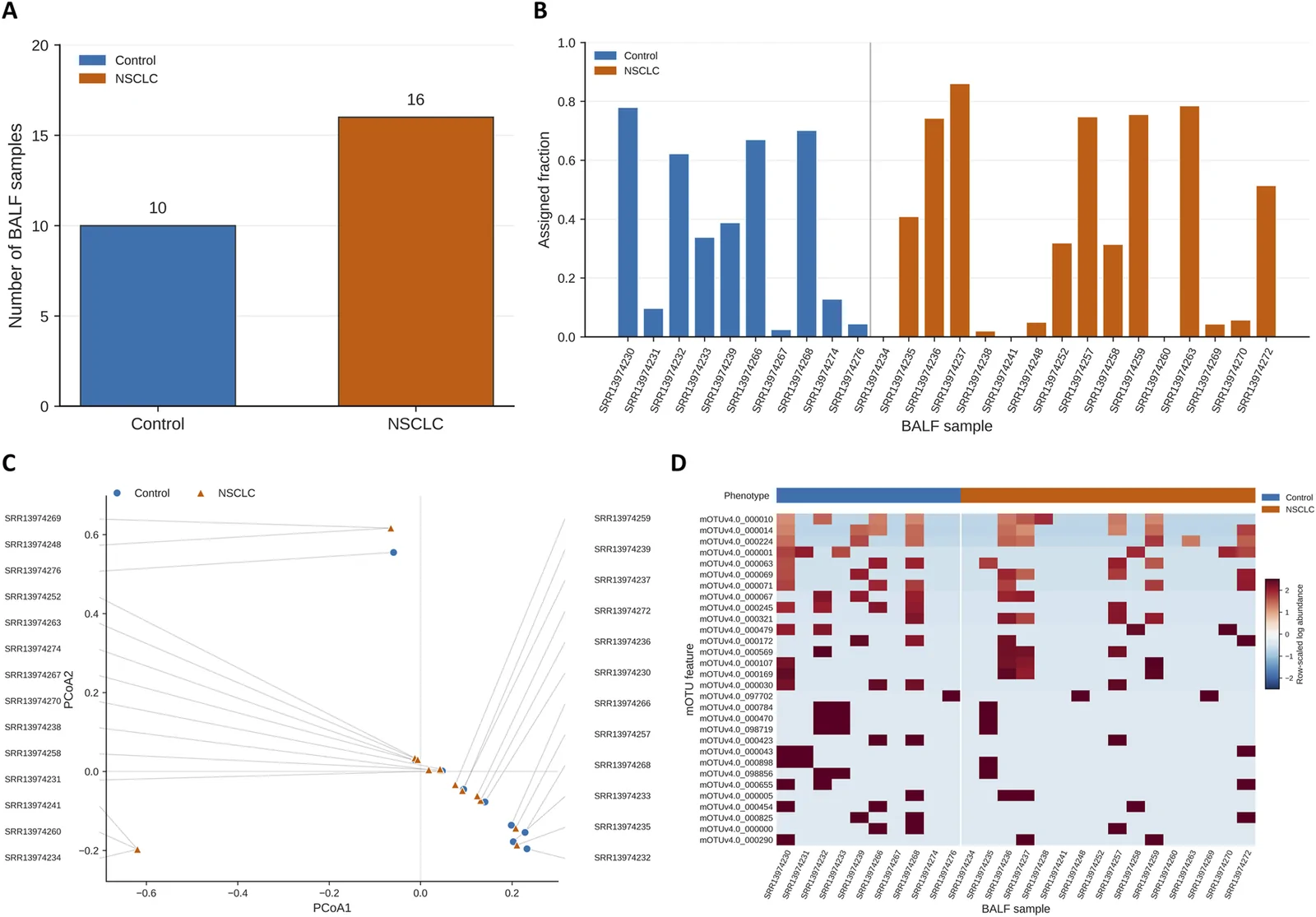
External application of LungMicroHostR to the PRJNA714488 BALF mOTU-level feature table. **(A)** Sample composition of the external dataset. **(B)** Sample-level assigned fraction from mOTUs v4 profiles; bars are ordered by phenotype and labelled with full SRR accessions. **(C)** Bray–Curtis principal coordinate analysis of assigned mOTU relative-abundance profiles; Control and NSCLC samples are distinguished by colour and point shape, and leader lines connect each point to its full SRR accession. **(D)** Heatmap of the 30 most prevalent assigned mOTU features; columns are BALF samples, rows are mOTU features, the colour scale shows row-scaled log abundance, and the annotation strip identifies Control and NSCLC samples.

LungMicroHostR processed the PRJNA714488 BALF shotgun metagenomic dataset as an external application example for microbial feature-table analysis (Figure 6). mOTUs v4 profiling produced an assigned mOTU-level feature table for 26 samples, including 10 controls and 16 non-small cell lung carcinoma samples. The external feature table contained 388 assigned mOTU features, and LungMicroHostR matched the feature table with phenotype metadata to generate a 388-feature by 26-sample abundance matrix.

The external analysis generated sample-level microbial assignment summaries, feature-prevalence tables, diversity summaries, Bray-Curtis ordination results and exploratory repeated stratified cross-validation outputs. The assigned fraction and mOTU feature summaries described sample-level microbial signal across the cohort (Figure 6B), while Bray-Curtis principal coordinate analysis and the heatmap of top prevalent mOTU features summarized variation across the external mOTU-level profiles (Figures 6C,D).

## Discussion

LungMicroHostR was designed for coordinated downstream analysis of microbial profiles, host-derived molecular measurements and negative-control information in respiratory mNGS data. Respiratory mNGS workflows can produce separate microbial and host feature tables, and interpretation requires sample-level matching before statistical analysis and visualization. Within this workflow, feature filtering, model evaluation and visualization are performed as connected steps in BALF mNGS host–microbiome analysis.

Existing microbiome software has greatly improved taxonomic profiling, feature-table handling, metadata analysis, statistical testing, visualization and microbial-community classification across diverse datasets (Table 1). The practical advantages of LungMicroHostR are its BALF-specific data structure and the coordinated handling of processed microbial and host-derived feature tables, sample partitions, negative-control screening flags, source-specific model comparisons, calibration and resampling outputs, and figure-ready result tables. Keeping these elements in one analysis record allows data matching, filtering decisions, model outputs and visual summaries to remain traceable within the same respiratory mNGS workflow. Feature tables generated by upstream profilers can therefore enter the same downstream structure at genus, species or mOTU level.

**TABLE 1:** Comparison of microbiome analysis tools and LungMicroHostR.

Tool/platform	Primary orientation	Typical input	Feature-table analysis	Association/biomarker testing	Classification/model evaluation	Host-feature or control-aware outputs	Representative References
QIIME 2	Microbiome data science platform	Amplicon/metagenomic feature data	Yes	Plugin/workflow dependent	Plugin/workflow dependent	User-defined workflow	Bolyen et al. (2019)
Phyloseq	R Infrastructure for microbiome data handling and visualization	OTU/ASV/taxonomic abundance tables with metadata	Yes	User-defined R workflow	User-defined R workflow	User-defined workflow	McMurdie and Holmes (2013)
MicrobiomeAnalyst/MicrobiomeAnalystR	Web and R-based microbiome statistical analysis and visualization	Microbial feature tables with metadata	Yes	Yes	Available modules	User-specified metadata	Dhariwal et al., 2017; Chong et al., 2020
Microeco/microeco 2	R-based microbiome community ecology and multi-omics data analysis	Microbial abundance tables, taxonomy tables, metadata and optional phylogeny	Yes	Yes	Workflow-dependent classification and machine-learning extensions	Metadata-aware ecological and statistical workflows	Liu et al., 2021; Liu et al., 2026
LEfSe	Microbial biomarker discovery	Taxonomic or functional abundance tables with class labels	Limited to biomarker workflow	Yes	No	No	Segata et al. (2011)
MaAsLin2	Multivariable association modeling for microbial meta-omics	Microbial features and sample metadata	Yes	Yes	No	Metadata covariates	Mallick et al. (2021)
SIAMCAT	Statistical inference and classification of microbial communities	Microbial feature tables with phenotype labels	Yes	Yes	Yes	Metadata-aware workflow	Wirbel et al. (2021)
mOTUs/MetaPhlAn	Shotgun metagenomic taxonomic profiling	FASTQ/shotgun metagenomic reads	Profiler output	No	No	No	Milanese et al., 2019; Beghini et al., 2021
LungMicroHostR	BALF mNGS downstream host–microbiome analysis	Microbial feature tables, host-derived features and sample metadata	Yes	Yes	Yes	Yes	This work

Abbreviations: ASV, amplicon sequence variant; BALF, bronchoalveolar lavage fluid; mNGS, metagenomic next-generation sequencing; OTU, operational taxonomic unit.

Method choice and preprocessing decisions can substantially influence microbiome results. Large-scale benchmarking analyses have shown that differential-abundance methods can produce different results on the same microbiome datasets, and that error control, confounding and data characteristics can influence performance (Nearing et al., 2022; Wirbel et al., 2024). Computational benchmarking guidelines also recommend prespecified scope, documented data and metric choices, and reproducible code and results (Weber et al., 2019; Mangul et al., 2019). LungMicroHostR follows these principles by keeping feature filtering, model evaluation and visualization within the same analysis workflow, so that filtering decisions and performance summaries remain connected during downstream analysis.

Negative-control prevalence is particularly relevant for BALF and other lower-airway specimens. Low-biomass microbiome data can contain background nucleic acids introduced during sampling, extraction, library preparation or sequencing, and background signals can affect diversity estimates, taxonomic composition and apparent disease-associated features when controls are overlooked (de Goffau et al., 2018; Karstens et al., 2019). Analyses of lower-airway samples have shown that laboratory contamination can alter inferred microbiome profiles, while accounting for taxa enriched in negative controls can improve comparability between datasets (Jervis-Bardy et al., 2015; Drengenes et al., 2019). In LungMicroHostR, prevalence in negative controls is stored as a transparent screening flag and is distinguished from formal contaminant-probability modelling. This makes recurrent background-prone features visible alongside the retained feature table during downstream analysis.

The public BALF mNGS analysis showed how model comparison can separate information carried by host-derived and microbial feature tables. Host transcriptomic features showed the strongest performance for distinguishing lung cancer from pulmonary infection in the test set, whereas RNA microbial features retained measurable discriminatory signal. The model combining host transcriptomic and RNA microbial features achieved balanced performance across several test-set metrics. This pattern indicates that host response captured by BALF mNGS is an important component of disease-state separation. RNA microbial signal may reflect infection-related organisms, active microbial transcription or host–microbial variation captured in BALF, and its contribution is evaluated alongside the host-derived reference within the workflow. The results also suggest that BALF mNGS data can be interpreted more completely when microbial profiles are considered together with host-derived molecular measurements.

The intended role of the modelling component is comparative evaluation across biological feature sources. Host transcriptomic models represent the host-derived reference, microbial-profile models represent microbial classification signal, and combined models evaluate host-derived and microbial features within the same framework. Its outputs summarize discrimination, threshold-based classification, calibration and uncertainty under a common protocol, clarifying whether performance is driven mainly by host-derived measurements, microbial profiles or their combination.

Separating model development from test-set assessment was central to the model comparison workflow. Feature filtering, model fitting and threshold definition were restricted to the training data, and final assessment was performed in the test set. The resulting comparisons considered separation between disease groups, agreement between predicted scores and observed outcomes, and stability of the estimates without relying on a single performance measure (Collins et al., 2015; Steyerberg, 2019).

LungMicroHostR maintains a consistent connection between data integration, statistical analysis and visualization. Statistical reporting and visualization are based on the same analysis results, reducing manual transfer between analysis scripts and graphical summaries. The analysis record therefore remains consistent from feature filtering to model evaluation and visualization (Sandve et al., 2013; Barker et al., 2022). This organization can make downstream results easier to review, repeat and adapt when similar respiratory mNGS feature tables are analysed.

The PRJNA714488 analysis provides an external application example for BALF shotgun metagenomic feature tables. By importing an assigned mOTU-level feature table and aligning it with phenotype metadata, LungMicroHostR generated sample-level microbial assignment summaries, feature-prevalence tables, ordination outputs and model-evaluation files. Together with the GSE252118 analysis, these results support LungMicroHostR as a flexible downstream framework for BALF microbial and host–microbiome data analysis.

Several limitations should be considered in relation to future development and broader use of LungMicroHostR. The 26-sample PRJNA714488 dataset was included as an external application example of mOTU-level feature-table processing and is interpreted as a compact workflow demonstration. Larger multicentre respiratory datasets will further expand benchmarking across sequencing platforms and analytical settings. Future releases can extend the framework to additional profiler-specific formats, functional profiles, strain-level summaries and automated supplementary-table exports.

## Conclusion

LungMicroHostR provides an R framework that connects microbial profiles, host-derived molecular measurements, negative-control information, model evaluation and visualization for downstream analysis of BALF mNGS host–microbiome data. In the public BALF mNGS analysis, host transcriptomic features formed the strongest reference signal, RNA microbial features retained discriminatory information and combined host–microbial models evaluated feature tables from different data layers within a common framework. The package provides a documented workflow for host–microbiome data integration, negative-control-based microbial feature screening and comparative model evaluation in respiratory mNGS analysis.

## Data Availability

The GSE252118 dataset is available in the Gene Expression Omnibus. The PRJNA714488 sequencing data are available in the NCBI Sequence Read Archive under BioProject PRJNA714488. The LungMicroHostR source code, documentation and examples are available from the GitHub repository: https://github.com/drleenan1988/LungMicroHostR. The revision reported here corresponds to commit 9e63dd860bb6da3263ef1b3cd787fe7e46aa752e.

## References

[B1] Barker M. Chue Hong N. P. Katz D. S. Lamprecht A. L. Martinez-Ortiz C. Psomopoulos F. (2022). Introducing the FAIR principles for research software. Sci. Data 9, 622. 10.1038/s41597-022-01710-x 36241754 PMC9562067

[B2] Beghini F. McIver L. J. Blanco-Miguez A. Dubois L. Asnicar F. Maharjan S. (2021). Integrating taxonomic, functional, and strain-level profiling of diverse microbial communities with bioBakery 3. eLife 10, e65088. 10.7554/eLife.65088 33944776 PMC8096432

[B3] Bolyen E. Rideout J. R. Dillon M. R. Bokulich N. A. Abnet C. C. Al-Ghalith G. A. (2019). Reproducible, interactive, scalable and extensible microbiome data science using QIIME 2. Nat. Biotechnol. 37, 852–857. 10.1038/s41587-019-0209-9 31341288 PMC7015180

[B4] Buza T. M. Tonui T. Stomeo F. Tiambo C. Katani R. Schilling M. (2019). iMAP: an integrated bioinformatics and visualization pipeline for microbiome data analysis. BMC Bioinforma. 20, 374. 10.1186/s12859-019-2965-4 31269897 PMC6610863

[B5] Cheng C. Wang Z. Ding C. Liu P. Xu X. Li Y. (2024). Bronchoalveolar lavage fluid microbiota is associated with the diagnosis and prognosis evaluation of lung cancer. Phenomics 4, 125–137. 10.1007/s43657-023-00135-9 38884058 PMC11169441

[B6] Chong J. Liu P. Zhou G. Xia J. (2020). Using MicrobiomeAnalyst for comprehensive statistical, functional, and meta-analysis of microbiome data. Nat. Protoc. 15, 799–821. 10.1038/s41596-019-0264-1 31942082

[B7] Collins G. S. Reitsma J. B. Altman D. G. Moons K. G. M. (2015). Transparent reporting of a multivariable prediction model for individual prognosis or diagnosis (TRIPOD): the TRIPOD statement. Ann. Intern. Med. 162, 55–63. 10.7326/M14-0697 25560714

[B8] Davis N. M. Proctor D. M. Holmes S. P. Relman D. A. Callahan B. J. (2018). Simple statistical identification and removal of contaminant sequences in marker-gene and metagenomics data. Microbiome 6, 226. 10.1186/s40168-018-0605-2 30558668 PMC6298009

[B9] de Goffau M. C. Lager S. Salter S. J. Wagner J. Kronbichler A. Charnock-Jones D. S. (2018). Recognizing the reagent microbiome. Nat. Microbiol. 3, 851–853. 10.1038/s41564-018-0202-y 30046175

[B10] Deek R. A. Ma S. Lewis J. Li H. (2024). Statistical and computational methods for integrating microbiome, host genomics, and metabolomics data. eLife 13, e88956. 10.7554/eLife.88956 38832759 PMC11149933

[B11] Dhariwal A. Chong J. Habib S. King I. L. Agellon L. B. Xia J. (2017). MicrobiomeAnalyst: a web-based tool for comprehensive statistical, visual and meta-analysis of microbiome data. Nucleic Acids Res. 45, W180–W188. 10.1093/nar/gkx295 28449106 PMC5570177

[B12] Drengenes C. Wiker H. G. Kalananthan T. Nordeide E. Eagan T. M. L. Nielsen R. (2019). Laboratory contamination in airway microbiome studies. BMC Microbiol. 19, 187. 10.1186/s12866-019-1580-1 31412780 PMC6694601

[B13] Eisenhofer R. Minich J. J. Marotz C. Cooper A. Knight R. Weyrich L. S. (2019). Contamination in low microbial biomass microbiome studies: issues and recommendations. Trends Microbiol. 27, 105–117. 10.1016/j.tim.2018.11.003 30497919

[B14] Fierer N. Leung P. M. Lappan R. Eisenhofer R. Ricci F. Holland S. I. (2025). Guidelines for preventing and reporting contamination in low-biomass microbiome studies. Nat. Microbiol. 10, 1570–1580. 10.1038/s41564-025-02035-2 40542287

[B15] Han D. Liu C. Yang B. Yu F. Liu H. Lou B. (2025). Metagenomic fingerprints in bronchoalveolar lavage differentiate pulmonary diseases. Npj Digit. Med. 8, 599. 10.1038/s41746-025-01977-5 41057624 PMC12504733

[B16] Jervis-Bardy J. Leong L. E. X. Marri S. Smith R. J. Choo J. M. Smith-Vaughan H. C. (2015). Deriving accurate microbiota profiles from human samples with low bacterial content through post-sequencing processing of illumina MiSeq data. Microbiome 3, 19. 10.1186/s40168-015-0083-8 25969736 PMC4428251

[B17] Karstens L. Asquith M. Davin S. Fair D. Gregory W. T. Wolfe A. J. (2019). Controlling for contaminants in low-biomass 16S rRNA gene sequencing experiments. mSystems 4, e00290. 10.1128/mSystems.00290-19 31164452 PMC6550369

[B18] Liu C. Cui Y. Li X. Yao M. (2021). Microeco: an R package for data mining in microbial community ecology. FEMS Microbiol. Ecol. 97, fiaa255. 10.1093/femsec/fiaa255 33332530

[B19] Liu B. Huang L. Liu Z. Pan X. Liu L. Wang D. (2022). EasyMicroPlot: an efficient and convenient R package in microbiome downstream analysis and visualization for clinical study. Front. Genet. 12, 803627. 10.3389/fgene.2021.803627 35058973 PMC8764268

[B20] Liu C. Li X. Mansoldo F. R. P. Chen T. Meng F. Tang R. (2026). Microeco 2: a comprehensive R package for downstream analysis of microbiome omics data. iMeta 5, e70132. 10.1002/imt2.70132 42491666 PMC13377413

[B21] Lu Y. Zhou G. Ewald J. Pang Z. Shiri T. Xia J. (2023). MicrobiomeAnalyst 2.0: comprehensive statistical, functional and integrative analysis of microbiome data. Nucleic Acids Res. 51, W310–W318. 10.1093/nar/gkad407 37166960 PMC10320150

[B22] Mallick H. Rahnavard A. McIver L. J. Ma S. Zhang Y. Nguyen L. H. (2021). Multivariable association discovery in population-scale meta-omics studies. PLoS Comput. Biol. 17, e1009442. 10.1371/journal.pcbi.1009442 34784344 PMC8714082

[B23] Mangul S. Martin L. S. Hill B. L. Lam A. K. M. Distler M. G. Zelikovsky A. (2019). Systematic benchmarking of omics computational tools. Nat. Commun. 10, 1393. 10.1038/s41467-019-09406-4 30918265 PMC6437167

[B24] McElderry J. P. Zhang T. Zhao W. Hoang P. H. Anyaso-Samuel S. Sang J. (2026). Microbiome analysis of 940 lung cancers in never-smokers reveals lack of clinically relevant associations. Nat. Commun. 17, 192. 10.1038/s41467-025-66780-y 41387456 PMC12780107

[B25] McMurdie P. J. Holmes S. (2013). Phyloseq: an R package for reproducible interactive analysis and graphics of microbiome census data. PLoS ONE 8, e61217. 10.1371/journal.pone.0061217 23630581 PMC3632530

[B26] Milanese A. Mende D. R. Paoli L. Salazar G. Ruscheweyh H. J. Cuenca M. (2019). Microbial abundance, activity and population genomic profiling with mOTUs2. Nat. Commun. 10, 1014. 10.1038/s41467-019-08844-4 30833550 PMC6399450

[B27] Nearing J. T. Douglas G. M. Hayes M. G. MacDonald J. Desai D. K. Allward N. (2022). Microbiome differential abundance methods produce different results across 38 datasets. Nat. Commun. 13, 342. 10.1038/s41467-022-28034-z 35039521 PMC8763921

[B28] Salter S. J. Cox M. J. Turek E. M. Calus S. T. Cookson W. O. Moffatt M. F. (2014). Reagent and laboratory contamination can critically impact sequence-based microbiome analyses. BMC Biol. 12, 87. 10.1186/s12915-014-0087-z 25387460 PMC4228153

[B29] Sandve G. K. Nekrutenko A. Taylor J. Hovig E. (2013). Ten simple rules for reproducible computational research. PLoS Comput. Biol. 9, e1003285. 10.1371/journal.pcbi.1003285 24204232 PMC3812051

[B30] Segata N. Izard J. Waldron L. Gevers D. Miropolsky L. Garrett W. S. (2011). Metagenomic biomarker discovery and explanation. Genome Biol. 12, R60. 10.1186/gb-2011-12-6-r60 21702898 PMC3218848

[B31] Shanmugam G. Lee J. Jeon J. (2021). EzMAP: easy microbiome analysis platform. BMC Bioinforma. 22, 138. 10.1186/s12859-021-04106-7 33827413 PMC8028061

[B32] Steyerberg E. W. (2019). Clinical Prediction Models: A Practical Approach to Development, Validation, and Updating. 2nd Edn. Cham: Springer.

[B33] Tang H. Liu H. Yuan L. Xie M. Zheng J. Wang J. (2025). Bronchoalveolar lavage fluid metagenomic datasets: a multidimensional clinical biomolecular resource. Sci. Data 12, 1919. 10.1038/s41597-025-06171-6 41350265 PMC12680720

[B34] Topçuoğlu B. D. Lapp Z. Sovacool K. L. Snitkin E. Wiens J. Schloss P. D. (2021). Mikropml: user-friendly R package for supervised machine learning pipelines. J. Open Source Softw. 6, 3073. 10.21105/joss.03073 34414351 PMC8372219

[B35] Weber L. M. Saelens W. Cannoodt R. Soneson C. Hapfelmeier A. Gardner P. P. (2019). Essential guidelines for computational method benchmarking. Genome Biol. 20, 125. 10.1186/s13059-019-1738-8 31221194 PMC6584985

[B36] Wirbel J. Pyl P. T. Kartal E. Zych K. Kashani A. Milanese A. (2021). Microbiome meta-analysis and cross-disease comparison enables microbiome-based diagnostics. mSystems 6, e00195-21. 10.1128/mSystems.00195-21

[B37] Wirbel J. Essex M. Forslund S. K. Zeller G. (2024). A realistic benchmark for differential abundance testing and confounder adjustment in human microbiome studies. Genome Biol. 25 (1), 247. 10.1186/s13059-024-03390-9 39322959 PMC11423519

[B38] Xu S. Zhan L. Tang W. Wang Q. Dai Z. Zhou L. (2023). MicrobiotaProcess: a comprehensive R package for deep mining microbiome. Innov. (Camb.) 4, 100388. 10.1016/j.xinn.2023.100388 36895758 PMC9988672

[B39] Zheng L. Sun R. Zhu Y. Li Z. She X. Jian X. (2021). Lung microbiome alterations in non-small cell lung cancer patients. Sci. Rep. 11, 11736. 10.1038/s41598-021-91195-2 34083661 PMC8175694

